# Uncovering the genomic landscape of *Mycobacterium bovis* in Wales

**DOI:** 10.1038/s41598-026-57014-2

**Published:** 2026-09-14

**Authors:** Amy J. E. Healey, Cate L. Williams, Nicholas J. Dimonaco, Terry Galloway, Richard J. Ellis, Eleftheria Palkopoulou, James Strong, Amanda J. Gibson, R. Glyn Hewinson, Jessica C. A. Friedersdorff

**Affiliations:** 1 Department of Life Sciences, Centre of Excellence for Bovine Tuberculosis, Aberystwyth University, Aberystwyth, Wales; 2 Institute for Global Food Security, School of Biological Sciences, Queen’s University Belfast, Belfast, Northern Ireland; 3 Animal and Plant Health Agency, Carmarthen Field Services, Ty Merlin, Heol Glasdwr, Parc Pensarn, SA31 2NF Carmarthen, Wales; 4 Surveillance and Laboratory Services Department, Animal and Plant Health Agency Weybridge, KT15 3NB New Haw, Surrey, UK; 5 Department of Bacteriology, Animal and Plant Health Agency Weybridge, KT15 3NB New Haw, Surrey, UK

**Keywords:** Bovine tuberculosis, MTBC, SNPs, Molecular epidemiology, Population structure, Wales, Computational biology and bioinformatics, Diseases, Genetics, Microbiology

## Abstract

Bovine tuberculosis (bTB), caused by the bacterium *Mycobacterium bovis*, is one of the most pressing animal health issues in Wales today. It negatively impacts cattle health, affects profitability and trade, and can decimate years of genetic improvement towards desirable production traits. It also imposes substantial financial, social, and psychological burdens on farming communities. Eradication of bTB requires an understanding of local transmission pathways to target effective disease-control interventions. In this study, we characterised the genomic diversity of *M. bovis* across Wales by analysing the genome sequence of 379 *M. bovis* isolates obtained from culture-positive animals in Wales in 2021. Analyses uncovered three prevalent clusters that are geographically distinct. A further three clusters containing fewer isolates were also geographically separated, two of which had particularly large SNP distances from most other Welsh isolates, suggesting independent introductions of *M. bovis* strains that are not endemic to Wales. Fine-scale and epidemiologically relevant genetic structuring was identified within the six main clusters, indicating region-specific evolution, which can drive local disease dynamics. Finally, SNPs were identified in coding genes that have the potential for important advantageous physiological consequences that may impact host-pathogen interactions and necessitate further investigation.

**Supplementary Information:**

The online version contains supplementary material available at https://doi.org/10.1038/s41598-026-57014-2.

## Introduction

*Mycobacterium bovis* is the primary pathogen that causes bovine tuberculosis (bTB) in cattle and zoonotic TB infections in humans^1^. Globally, *M. bovis* is found to be maintained in several different wildlife reservoirs beyond cattle, specifically in Wales the European badger (*Meles meles*) is a significant wildlife reservoir^2^ which adds complexity to bTB eradication strategies. Bovine tuberculosis is an economically significant disease and is endemic amongst animal hosts in the UK. Whilst Scotland has achieved bTB-free status, recent statistics^3,4^ show that although there has been a significant decrease in incidence since 2010 (-8.6-6.3%), in Wales bTB remains a persistent and endemic disease, with annual prevalence of 5.7% in 2024. In England and Wales, the estimated mean total consequential cost per breakdown is £23,636^5^, but the social and psychological implications for farmers and herd managers are currently impossible to quantify^6^.

*M. bovis* belongs to the Mycobacterium tuberculosis complex (MTBC), alongside at least ten other lineages that cause similar respiratory symptoms in a variety of hosts, including humans. MTBC is a clonal complex, thought to have originated from a single common ancestor with members collectively sharing 99.95% genetic identity^7–9^. Horizontal gene transfer (HGT) and recombination events are rare within MTBC; therefore, due to the clonal nature of the MTBC, almost all variation is observed through single nucleotide polymorphisms (SNPs) and insertion/deletion events (indels), most of which are located in repeat regions^9,10^. *M. bovis* belongs to one of the animal-adapted lineages of the MTBC. There have been several key phylogenetic investigations that have organised *M. bovis* into four (EU1, EU2, AF1 & AF2) defined clonal complexes^11^ or at least eight (La1.1-1.8) lineages^12^ that are geographically separated globally. According to Zwyer et al.^12^, four of these lineages are organised into monophyletic groups corresponding to the major clonal complexes: EU1 is associated with La1.8.1, EU2 with La1.7.1, AF1 with La1.6, and AF2 with La1.3. The remaining lineages represent distinct genetic groups. The complex EU1/lineage La1.8.1 accounts for almost all animal infections in the UK and has also been identified in several countries worldwide^13,14^.

Whole genome sequencing (WGS) has superseded traditional typing methods to characterise *M. bovis* isolates and for molecular epidemiological studies^15^. Studies using WGS in conjunction with traditional typing methods, such as spoligotyping and variable-number tandem of repeats (VNTR), have found that SNP-based phylogenies derived from WGS data separate samples geographically and reveal strong local dynamics^15,16^. As such, SNP patterns, when used in combination with existing molecular methods or independently, have been shown to provide greater epidemiologically relevant granularity than traditional methods. For example, genotypic differences between *M. bovis* isolates from outbreaks of bTB from across the USA^17^ and UK^15^ were resolved using SNP patterns, where clades defined by SNP data were represented by more than one spoligotype/VNTR profile, demonstrating the ability of SNP-based phylogeny to provide higher resolution than traditional genotyping methods.

The localisation of SNPs across the genome may also have the potential for functional impact. Previous analysis of *M. bovis* isolates using WGS revealed that of all SNPS occurring within virulence genes, 33% of these SNPs occurred in genes associated with lipid transport and metabolism^18^. Perhaps not surprisingly, given the importance of the lipid cell wall and its role in virulence and interactions with host immune cells, these genes are also subject to heavy selection pressure by the diagnostic skin test, specifically the single intradermal comparative cervical skin test (SICCT)^19^. It is therefore essential to provide a functional context for the SNPs used to genotype *M. bovis* strains.

Recently, the Animal Plant Health Agency (APHA) replaced genotyping of field isolates by spoligotyping and VNTR analysis^20,21^ with the use of WGS for *M. bovis* isolates cultured from lymph nodes of reactor animals in England and Wales, and have recommended a shift to a SNP-based classification system to replace the genotype scheme that has been used previously^15^. In this study, we complement the annual APHA bTB report from 2021^3^, which contains descriptive epidemiology reports on bTB in Wales by analysing the genome sequence of all *M. bovis* isolates obtained in Wales in 2021 to elucidate the population structure of *M. bovis* in Wales and assess the distribution and potential effects of SNPs in these isolates.

## Results

A total of 477 samples were available for this study from Wales in 2021, each consisting of paired-end reads sequenced from a putative *M. bovis* sample recovered from predominantly bovine hosts, as well as from badgers (*N* = 10) from across Wales, fallow deer (*N* = 2) isolated from deer in Carmarthenshire, and a single sample isolated from a TB positive cat located in Glamorgan. Of the 477, 98 samples failed the coverage filtering step, resulting in a total of 379 samples. A total of 2,047 SNPs were identified across all 379 samples across Wales.

### Phylogenetic analysis reveals *M. bovis* population structure in Wales

**Fig. 1:**
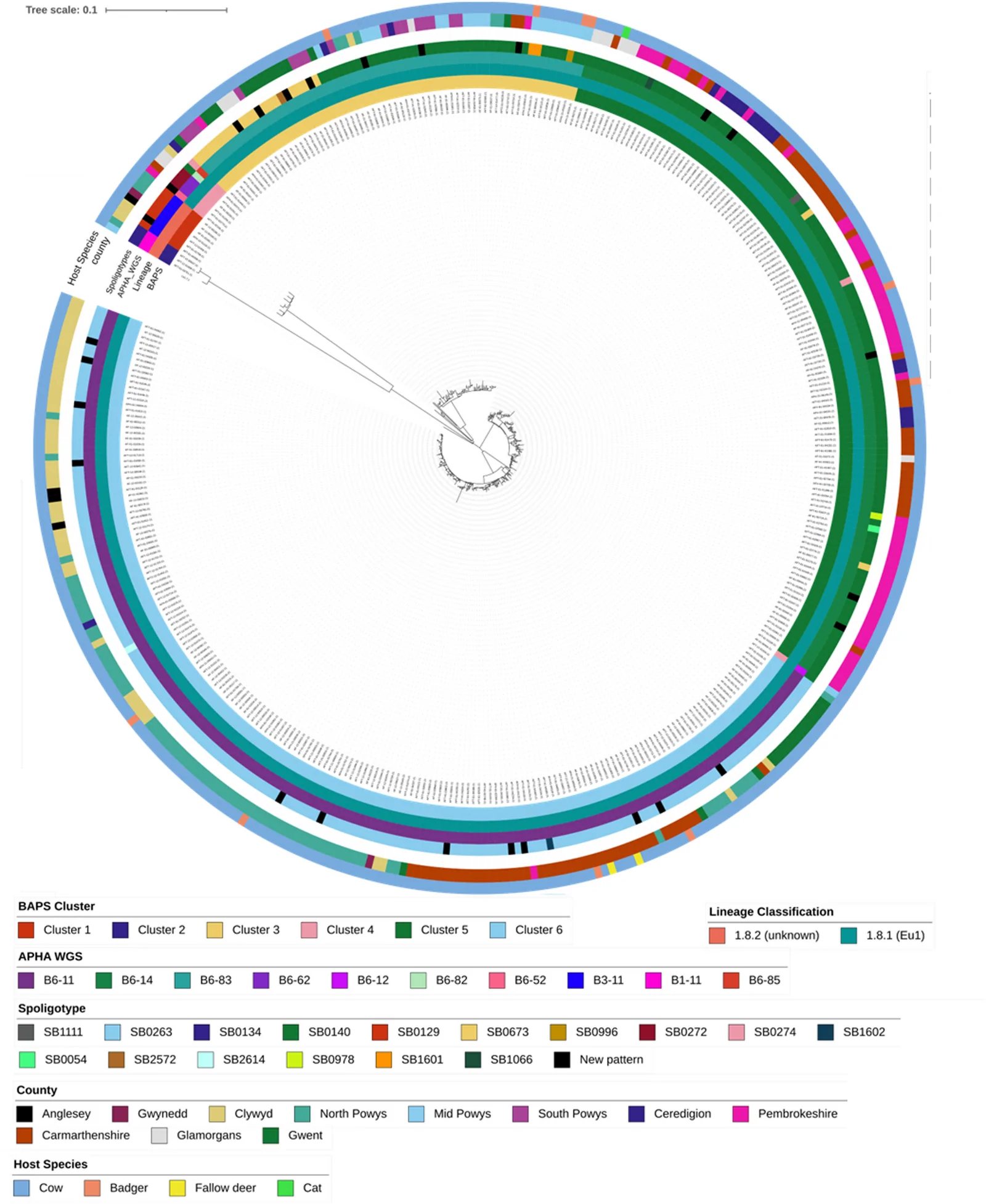
Maximum likelihood phylogenetic tree of *M. bovis* inferred from 1,971 SNPs from 379 isolates across Wales. Lineage La1.7.1 of Zwyer et al.^12^ was used to root the tree. The phylogeny is annotated with coloured concentric rings; from inside out, these represent (1) level one BAPs cluster, (2) the lineage of Zwyer et al.^12^ that isolates were assigned to, (3) the APHA WGS clade of Sandhu et al.^15^ that isolates were assigned to, (4) the spoligotype of the isolate, (5) the Welsh county the sample was obtained in, and (6) the host species the sample was obtained from. Trees were constructed using IQTree and the TVM + F+I + I+R7 model.

After filtering of SNPs within 10 bp of each other, a subset of 1,971 SNPs (of a total of 2,047) were used to create phylogenies and for further downstream analyses. Fast-BAPS Bayesian clustering (innermost ring in Fig. 1) partitioned the dataset into six distinct clusters, which was supported by Maximum likelihood (ML) phylogenies; with bootstrap (BS) values of 100 for all clusters, excluding BAPs assigned cluster 4; which was paraphyletic in the ML phylogeny.

Samples were assigned to the bTB lineage classification of Zwyer et al.^11^ (Fig. 1), with all samples falling within lineage La1.8.1, except for those in clusters 1 and 2, which were assigned to La1.8.2. Samples were also assigned to the APHA WGS clades (Fig. 1 & Supplementary Fig. 1) defined in Sandhu et al.^15^, and the BAPS clusters defined here corresponded to monophyletic APHA WGS clades (excluding cluster 4). Spoligotypes (Fig. 1) were also determined for all 379 samples and were near-completely aligned with BAPS clustering.

**Fig. 2:**
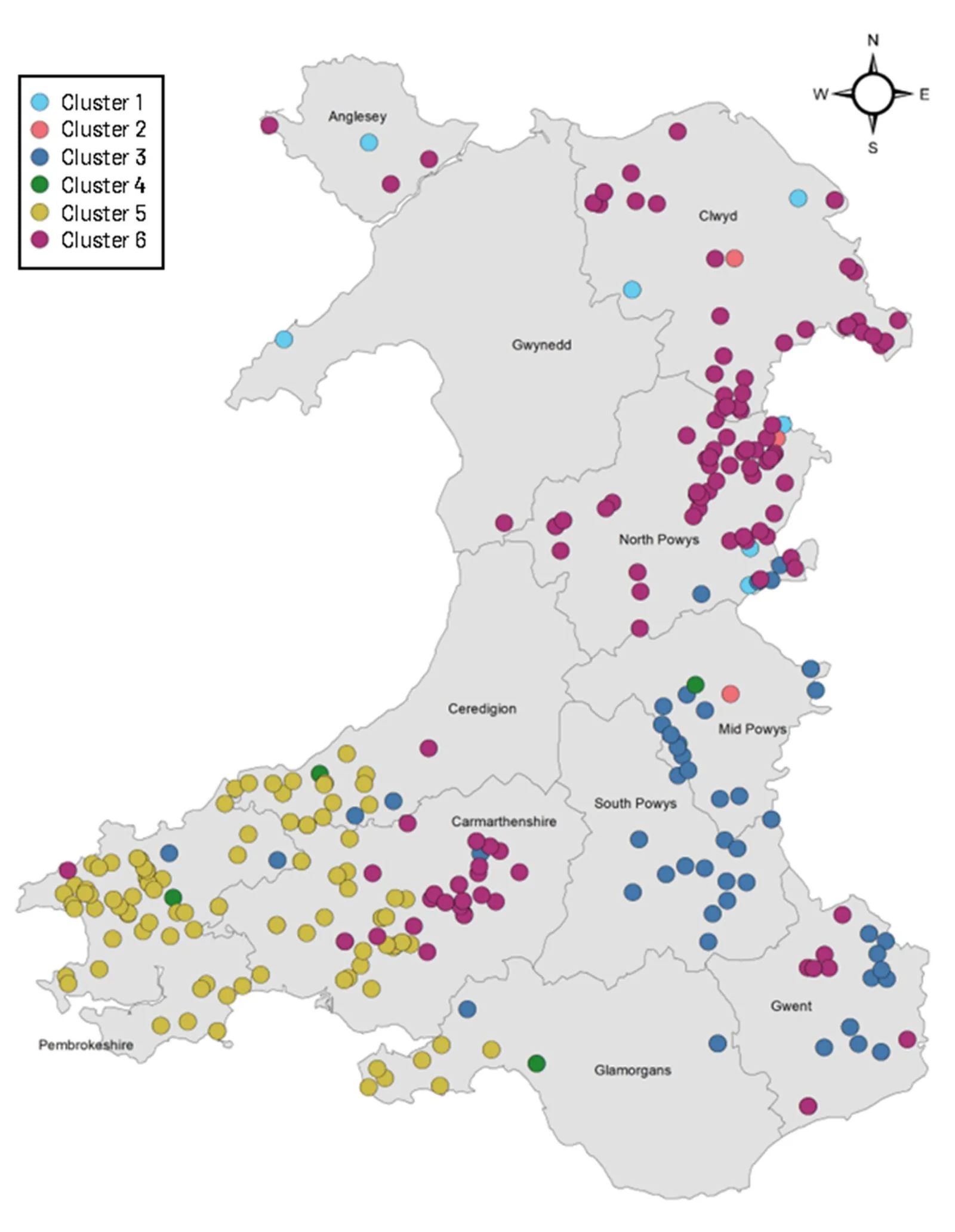
Map of sample locations across Wales. Sampling points are coloured to represent the membership of each sample to each of the six genetic clusters identified by BAPS analysis, where light blue is cluster 1, pink is cluster 2, dark blue is cluster 3, yellow is cluster 5 and purple is cluster 6. Note that the county Powys has been split into three for ease of discussion. Map was created in ESRI ArcGis 10.2.2 (www.esri.com/en-us/arcgis).

Some geographic localisation of clusters was evident (Fig. 2), particularly for clusters 3, 5 and 6. Each of these clusters could be readily aligned with one of the WGS clades defined by APHA^15^ (Fig. 1 & Supplementary Fig. 1), with Cluster 1 = B3-11, Cluster 2 = B1-11, Cluster 3 = B6-83, Cluster 5 = B6-14 and Cluster 6 = B6-11, except for cluster 4, which aligned with multiple APHA WGS clades (B6-12, B6-52, B6-62, B6-82 & B6-85). This indicates that cluster 4 comprises multiple distinct genetic subgroups that were not assigned as distinct clusters by fastBAPs partitioning, this is further supported by the greater within-cluster SNP differences resolved in cluster 4 (Supplementary Tables 1 and 2, Supplementary Fig. 3). As the paraphyly of cluster 4 is likely to skew any comparisons, cluster 4 is not discussed further in regard to population structure or functional mutations, although it is included in downstream analyses.

The average SNP distance (Supplementary Tables 1 and 2, Supplementary Fig. 2) between clusters ranged between 122 (stdev = 4.51) and 516 (stdev = 8.07) SNPs, with clusters 1 and 2 the most distinct, differing from all other clusters by 406–475 and 474–505 SNPs, respectively. All the other clusters (3–6) differed from each other by fewer SNPs (122–160). The diversity within clusters (Supplementary Table 3) varied.

**Fig. 3:**
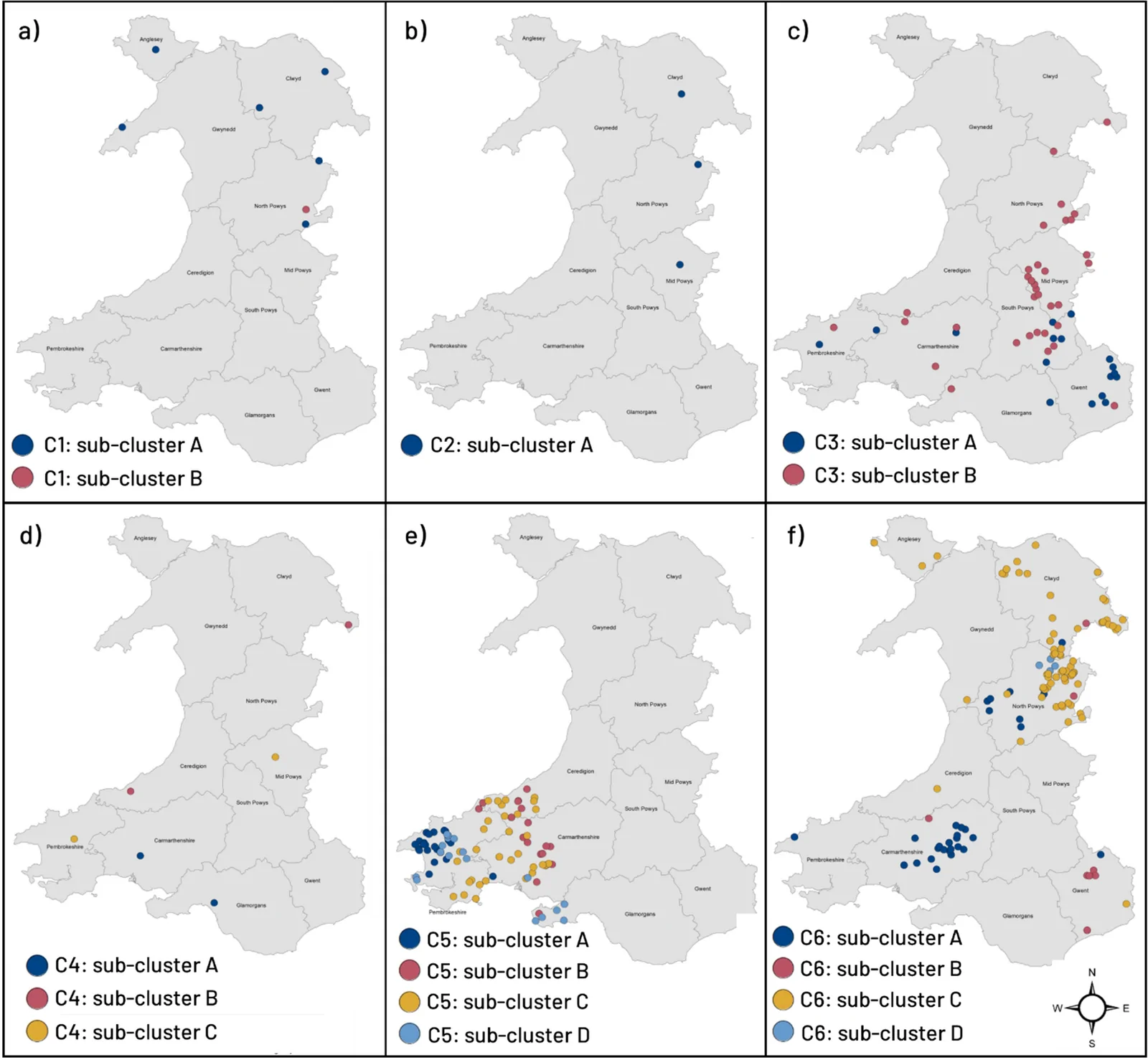
Maps of the distribution of each of the clusters identified by BAPS analysis. (**a**) Cluster 1, (**b**) Cluster 2, (**c**) Cluster 3, (**d**) Cluster 4, (**e**) Cluster 5, (**f**) Cluster 6. Sample points are colour coded based on membership to each of the within cluster (C1-C6) subclusters defined by level 2 BAPS analysis. Maps were created in ESRI ArcGis 10.2.2 (www.esri.com/en-us/arcgis).

Within the six main clusters, further within-clade clustering of isolates was observed using the fastBAPS algorithm (Supplementary Fig. 3) and supported by Maximum Likelihood (ML) BS values of 100 for the nodes separating each monophyletic sub-cluster in the ML-derived phylogeny. When the locations of these sub-clusters were mapped (Fig. 3), geographically localised sub-clustering was resolved within clusters 3, 5 and 6.

### SNP effects on function

**Fig. 4:**
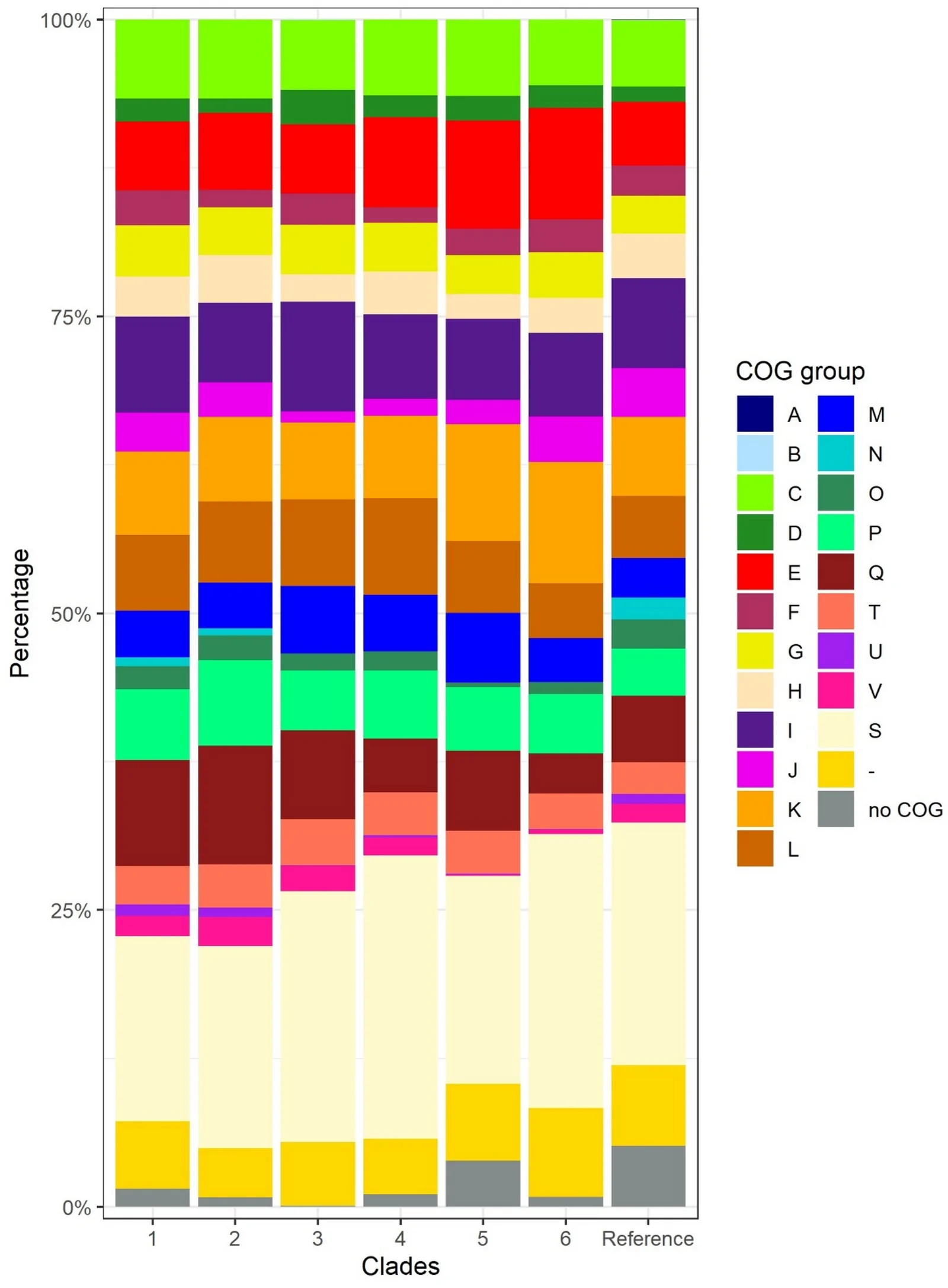
Stacked bar graph with the functional breakdown of SNPs in genes that belong to different COG groups for the six clusters. The proportion of genes belonging to the different COG categories in the reference genome is given for context.

Of the 2,047 variants identified across all samples, 1,770 SNPs occurred in protein coding regions, 271 in non-coding regions and a further six occurred in ribosomal RNA regions (Supplementary Table 5). There were three SNPs that occurred in the overlapping region between two genes (both genes are counted separately in the functional analysis). There were 651 SNPs that resulted in synonymous mutations, and 1,120 in non-synonymous (1,104 missense and 16 nonsense) mutations, giving a ratio of 0.58 synonymous to non-synonymous SNPs (dN/dS = 1.72). Generally, no single cluster showed a bias towards an accumulation of SNPs in any one functional group (Fig. 4; Supplementary Table 4).

Based on *M. bovis* essentiality allocations^22^, a total of 1,354 SNPs were identified in 958 non-essential (NE) genes, 225 SNPs in 166 essential genes, 48 SNPs across 38 genes that confer a growth advantage and 143 SNPs across 87 genes that confer a growth disadvantage. Of the essential genes with SNPs, those genes involved in intermediary metabolism and respiration harboured the highest percentage of SNPs at 34%. Around 60% of SNPs in essential genes were missense mutations, the remainder were silent. There was one nonsense mutation in a conserved protein (Mb0504c; orthologous to Rv0493c^23^) that was present in seven isolates. In addition, there was one missense mutation found in 10 samples that was marked as high impact and results in a loss of a stop codon in a probable ABC transporter protein (Mb1013; orthologous to 5’ end of Rv0987). Out of 181 virulence genes listed previously^23^, 58 genes contained a total of 114 SNPs. Of these, three SNPs were reported as high impact as the SNP causes a stop gain in genes *plcD* (Mb1784c), *ribA1* (Mb1975, ortholog Rv1940), and *lipR* (Mb3111; ortholog Rv3084). A further 67 SNPs were missense mutations in 41 virulence genes, and 44 were silent mutations in 23 virulence genes.

No SNPs were found in any samples in the genes encoding the antigens ESAT-6 (*esxA*, Mb3905), CFP-10 (*esxB*, Mb3904), and Rv3615c (*espC*, Mb3645c), nor were any SNPs found in genes encoding the sero-diagnostic antigens MPB70 (Mb2900) and MPB83 (Mb2898). When normalised for gene length, the gene with the highest frequency of SNP mutations was a conserved hypothetical protein (Mb0772) with 0.0122 SNPs/BP. There were 351 genes containing more than one SNP.

There were 25 SNPs tagged as high impact by SnpEff, which caused start loss (*n* = 4), stop gain (*n* = 16) or stop loss (*n* = 6). All but two of the high-impact SNPs were found in non-essential genes. One SNP which causes the loss of a stop codon in a probable FadB3a protein (Mb1742; ortholog Rv1715) occurred in 378 samples. In 46 isolates (belonging to Cluster 5 subcluster C), a stop codon was gained in a transcriptional regulator of the AcrR family (Mb0848c; ortholog Rv0825c). These 46 isolates all belong to Cluster 5 (Supplementary Fig. 4) and show geographical localisation; found in Carmarthenshire, south Ceredigion, and Pembrokeshire.

There were 10 isolates together from Cluster 1 (*n* = 7) and Cluster 2 (*n* = 3) that all contained the same four high-impact missense SNPs, which were unique to these two clusters, each predicted to result in the loss of a stop codon from that gene (Mb0731, Mb1013, Mb1351c, Mb2607). An additional four high-impact SNPs were unique to Cluster 1, three of which result in premature stop codons and one in the loss of a start codon. Another two SNPs were unique to Cluster 2, all of which resulted in a premature stop codon (Supplementary Fig. 4).

## Discussion

This study utilised WGS data for all *M. bovis* isolates collected across Wales in 2021 from infected cattle (and from badgers, fallow deer and a cat) to define the population structure of *M. bovis* in Wales. These analyses complement the annual bTB epidemiology and surveillance reports published by APHA^3^, providing fine-scale spatial resolution for Welsh *M. bovis* isolates. Furthermore, WGS data were utilised to identify functionally significant SNP mutations throughout the phylogeny that could have advantageous physiological effects.

### Population structure of Welsh *Mycobacterium bovis*

WGS data provided considerable resolution for evaluating population structure of epidemiological relevance, and sheds light on the physical and evolutionary factors that shape the distribution and abundance of *M. bovis* in Wales. The genome-wide SNP patterns partitioned the *M. bovis* isolates in the 2021 dataset into six distinct clusters, of which three groups were dominant: Cluster 3 (17.1% of all Welsh samples), Cluster 5 (31.1%), and Cluster 6 (47.1%). In addition to these three main genetic lineages, three less common genetic clusters (Clusters 1, 2 & 4) were identified. Both Clusters 1 and 2 were highly genetically divergent from the rest of the Welsh *M. bovis* population. These genetic differences, as well as phylogenetic partitioning, suggest that Clusters 1 and 2 have been diversifying along their own evolutionary trajectories for a significant amount of time and originate from different ancestral lineages than the *M. bovis* reference strain, AF2122/97, which is assigned the EU1 clade/lineage La1.8.1, the most common genetic group in the UK. Isolates in clusters 1 and 2 were assigned to WGS lineage La1.8.2 (11) and spoligotypes SB0129 and SB0134, respectively, which have been shown to be more closely related to French strains^24–26^, and recognised as representing a distinct clonal complex^11,27^ supporting the hypothesis of an alternate ancestor to the reference strain. The small number of isolates identified within Clusters 1 and 2 suggests that these are uncommon genotypes in Wales.

There was a clear geographic basis to the distribution of the three dominant genetic types across Wales; Cluster 3 was the dominant genetic lineage in mid and southeast Wales, Cluster 5 was found only in southwest Wales, and Cluster 6 was found primarily in northeast Wales, as well as in three localised and spatially aggregated clusters in Carmarthenshire, Anglesey and Gwent. These results are consistent with the WGS clade home ranges for Wales reported recently by Seery et al.^4^ and align with the results of Sandhu et al.^15^, which identified B6-11 (cluster 6) as the most prevalent and widespread clade in the UK. The home ranges for B6-11 (cluster 6) and B6-83 (cluster 3) extend across the border of Wales into west and south-west England^4,15^, whilst B6-14 (cluster 5) is unique to Wales, with all cases elsewhere attributed directly to cattle movement from southwest Wales (APHA personal communication).

By comparing the genetic structure resolved here with that of previous studies, we can infer how the spatial distribution and abundance of genetic types in Wales have changed over time; however, to compare the genomic signatures reported here with historical population dynamics (which pre-date the widespread WGS monitoring of *M. bovis*), signatures of spatio-temporal genomic differentiation must be interpreted in relation to the spoligotype that they are assigned to. In 2003, the SB0140 spoligotype (which corresponds with Cluster 3 Subcluster B/B6-83 and Cluster 5/B6-14) was reported as the most abundant spoligotype in Ceredigion, Carmarthenshire, Pembrokeshire and Powys, and was present in Gwent, (as well as being the most common spoligotype across the whole of the UK)^28^. This remains true in the present study, however, SB0140 is polyphyletic and has been observed in multiple WGS clades across the UK^15^; therefore, the historical abundance of this spoligotype likely reflects the presence of multiple distinct WGS clusters. Spoligotype SB0263 (Cluster 6/ B6-11) has shown a recent population expansion; it was previously the second most common in the UK^28^ and has since become the most abundant genetic group^15^, accordingly it was the most common and geographically dispersed genetic group in west and south-west England^15^ and the most common genetic group reported across Wales in this current study.

Such region-specific evolution and potential adaptation can drive local disease dynamics and outcomes and have been well documented for *M. tuberculosis* infection in humans^29–31^. The fine-scale granularity of variation resolved here offers the potential to improve our understanding of the origin of infection and associated local risk pathways for the spread of bTB. This knowledge will help target optimal control interventions, which will be the focus of future studies.

### The role of cattle movement and wildlife reservoirs in shaping population structure

The distribution and abundance of *M. bovis* isolates in Wales generally reflect the density of cattle herds, with more samples in Pembrokeshire, Carmarthenshire, and north Powys, and fewer occurrences in the central mountainous regions, where cattle density is lower. A notable exception to this is Anglesey and the Llyn Peninsula, where, despite a high cattle density comparable to the higher TB areas of Pembrokeshire and north Powys, the prevalence of *M. bovis* is low. The density of hosts is recognised as being of critical importance to the transmission of bTB^32–34^, which necessitates careful consideration in terms of the management of bTB; particularly given the trend towards intensification of farming practices over recent decades^35^. This highlights the importance of a ‘keep it out’ policy for areas of Wales with a low incidence of *M. bovis* infection and for herds free of tuberculosis.

Cattle movement is well recognised as a major driver of *M. bovis* transmission across long distances^27,36–39^, and the phylogenies reported in this study, when interpreted in the context of reported cattle movement data, suggest cattle movement may have played an important role in introducing many of the genetic clusters present in Wales. For example, isolates with distinct genetic types, separated by large SNP differences from the other clusters, may indicate an imported strain. Isolates in Clusters 1 & 2 belong to the La1.8.2 lineage of Zwyer et al.^11^, and exhibit the SB0129 and SB0134 spoligotypes, respectively. These genetic groups have been identified in countries worldwide, including South Africa, Madagascar, Spain, Mali, France, and Brazil^11,24,40^, and are the most prevalent genetic types reported in Ethiopia^41^ and Algeria^42^. Isolates belonging to this La1.8.2 lineage are distinct from those of the GB-dominant EU1 clonal complex^11^. This suggests that isolates with this spoligotype and thus the La1.8.2 lineage may have been imported, though whether this is multiple independent import events into GB or a single introduction followed by population expansion via intra-GB movement requires further investigation. Samples with the SB0134 spoligotype have previously been recorded in isolates from Dyfed, Gwent and Powys, as well as in the English counties of Gloucestershire, Herefordshire, Worcestershire, and Cornwall^11^. The corresponding APHA WGS clades (B1-11 & B3-11) are commonly identified in the English counties bordering Wales^21^ which suggests that these lineages are well established within the UK. As such, although we cannot rule out international import, the presence of these lineages in Wales is most likely attributed to more local movement between England and Wales.

Within-cluster phylogenetic partitioning provides further evidence for the role of cattle movement in transmitting distinct genetic lineages across Wales, and sheds light on the demographic events that occur post-transmission. Cluster 6 (B6-11) has undergone recent spatial expansion and proliferation in northern Wales^3,4^, with increasing frequency in northeast Wales, and has extended its range into Anglesey and Gwynedd between 2019 and 2020. Furthermore, the intra-clade genetic structure resolved here indicates the presence of two founder events^43^ of this genetic lineage into Carmarthenshire (Cluster 6: Sub-cluster B) and Gwent (Cluster 6: Sub-cluster A), which were most likely introduced by the movement of infected cattle into these regions from the historical home range of the respective genetic type. This was then followed by rapid population expansion^27,44^, which can be associated with efficient transmission amongst cattle in these regions. Further analyses of temporal variation in these genetic sub-clusters across Wales are required to better understand the drivers and dynamics of these localised outbreaks.

In the British Isles, the Eurasian Badger (*Meles meles*) acts as a wildlife reservoir for maintaining and spreading *M. bovis* infection^45^. Although the badger data reported here are limited, the isolates do not show host specificity, as demonstrated by a lack of clustering by host species in the phylogenetic tree. Instead, bTB isolates from badgers are most closely related to isolates from sympatric cattle, consistent with previous observations from an all-Wales Badger Found Dead survey carried out between 2014 and 2016^46^. Further investigations are required to understand the local drivers of *M. bovis* transmission amongst hosts.

### The functional potential of SNPs in *M. bovis* in Wales

In total, 1,770 SNPs were found in protein-coding genes. A ratio of synonymous to non-synonymous SNPs of 0.58 was calculated for this dataset, consistent with previously reported values for *M. bovis* isolates^17,24^. The number of missense mutations was approximately twice that of silent mutations, with very few nonsense mutations. Missense mutations result in an amino acid change, which may affect protein structure and subsequently function, but equally may not affect the protein sequence; as such, it is difficult to predict the impact of such SNPs^47^. Nonsense mutations, however, are more likely to alter protein structure and thus function by introducing a premature stop codon. Of particular interest would be premature stops occurring in regulators, which will impact the expression of one or more genes within a regulon. Such might be the case with the transcriptional regulator gene in the AcrR family (Mb0848c), where a SNP introduces a premature stop codon, and this mutation occurs in all 46 isolates belonging to Cluster 5, Subcluster 3. To assess whether this SNP confers an advantage, temporal data are needed to determine whether any population expansion is associated with a competitive advantage. Additionally, in vitro studies are required to determine its phenotypic effects, alongside a combination of in vitro and in vivo studies to evaluate its immunological impact on the host.

Across the six clusters, no single Clusters of Orthologous Genes (COG) category contained significantly more SNPs than the others. A hypothesis explored here was whether SNPs congregate in genes with similar key functions (such as lipid metabolism) that together improve the success of *M. bovis* isolates and that these advantageous mutations would form a distinct clade. This, however, was not the case here. Overall, the COG category containing the most SNPs was transcription (K) (excluding genes with unknown function), which is also the functional group with the most genes in the reference genome. This has been highlighted by Bigi et al.^48^, who found variations in 20 regulatory proteins in *M. bovis* compared to *M. tuberculosis* and postulated that these may contribute to host specificity and influence behaviour under hypoxic environments. This may reflect the generalised enrichment of bacterial genomes with transcription factors, which enables rapid adaptation to complex environmental conditions through the regulation of gene expression^49–51^. Furthermore, the lack of SNPs in core virulence factors ESAT-6, CFP-10, and EspC highlights their high degree of conservation, as described in other literature^52^, and reinforces the utility of these antigens in the diagnosis of bTB using either skin tests or gamma interferon-based diagnostic assays.

SNPs were identified in 89 of the 181 virulence genes described previously in *M. bovis*^24^. Of interest are those highlighted as high impact due to nonsense mutations. The *plcD* gene (Mb1784c) is unique to *M. bovis* and has no ortholog in *M. tuberculosis* H37Rv; it is a membrane-bound phospholipase C and, in a very small number of *M. bovis* isolates, is also disrupted by transposition of the IS6110 element^53,54^. Yet, despite the well-known role of phospholipase C proteins in virulence in several intracellular pathogenic bacteria (including *M. tuberculosis*) its precise role in *M. bovis* is yet unknown^54^. The gene *ribA1* (Mb1975) is involved in the riboflavin biosynthesis pathway, where *ribA1* and *ribA2* catalyse the first step in the chain reaction breaking down GTP^55^. The introduction of a premature stop codon and effective knock-out of the gene would not impact riboflavin biosynthesis, possibly due to the compensatory activity of *ribA2*, supported by the classification of *ribA1 *as NE in *M. bovis*^22^. The *lipR *gene (Mb3111) encodes an esterase that hydrolyses short-chain esters. In *M. tuberculosis* the esterase was identified in inclusion bodies, and mouse models revealed its ability to inhibit interferon-γ (IFN-γ) and interleukin-2 (IL-2) secretion and stimulation of IL-10^56^. The study by Chun-Xi et al.^56^ demonstrates anti-inflammatory activity via the inhibition of pro-inflammatory cytokines and suggests that this protein plays a role in host-pathogen interaction, at least in *M. tuberculosis*. The introduction of a premature stop codon early in the sequence (base 149/927) would likely knock out the gene, a phenomenon that is frequently seen in *M. tuberculosis* where the gene contains large sequence polymorphisms resulting in complete deletion of *lipR* in clinical isolates from patients with active disease^57^. This suggests that the SNP in Mb3111 may not significantly affect virulence or transmission but may alter the expression of some cytokines in the host during infection.

### Not all M. bovis isolates have split proteins

Cluster 1 and 2 are distinct from the other clusters observed in this study (406–516 SNP difference between Clusters 1 and 2, and all other clusters). Isolates in these clusters also possess more unique SNPs with the potential to affect functional changes, as identified by SnpEff as high impact. Some of these SNPs appear to cause the loss of stop codons present in the genomes of *M. bovis* AF2122/97 and members of clusters 3, 4, 5 and 6. Orthologous genes in *M. tuberculosis*, (which shares a common ancestor with *M. bovis*^58^), encode fully functional proteins in these differential regions. It is the acquisition of a SNP in the evolution of *M. bovis* that results in a premature stop codon and the splitting of these proteins. There are four examples in the present dataset where split proteins are created by the presence of a stop codon in *M. bovis* clusters 3–6. The gene *atsA *(Mb0731; ortholog Rv0711) encodes an arylsulfatase that exists as a single protein in *M. tuberculosis* H37Rv, whereas in *M. bovis*, this gene is split into* atsA* and *atsB*^23^. For the *M. tuberculosis* H37Rv gene Rv0987 that encodes an ABC transporter, the stop codon in *M. bovis* results in the protein being split into Mb1013 and Mb1014. For *alkA*, a methyltransferase encoded by the singular Rv317c in *M. tuberculosis* H37Rv, the premature stop codon results in alkAa (Mb1351c) and alkAb (Mb1350c) in *M. bovis*. Finally, a SNP in *M. bovis* causes a frameshift in the single conserved hypothetical protein Rv2577 in *M. tuberculosis* H37Rv, resulting in Mb2607 and Mb2608^23^. The 10 isolates belonging to Clusters 1 and 2 possess the same alleles for these four genes as *M. tuberculosis* H37Rv, suggesting that perhaps not all *M. bovis* isolates possess split proteins. This evidence suggests that isolates belonging to Clusters 1 and 2 may have descended from an ancestor other than *M. bovis* AF2122/97, one that originated before the acquisition of the SNPs that gave rise to the split proteins.

## Conclusion

In this study we characterised the genomic diversity of *M. bovis* across Wales by analysing the genome sequence of all 379 *M. bovis* isolates obtained in Wales in 2021. The use of SNPs identified in *M. bovis* isolates provided greater resolution to traditional methods, such as spoligotyping and VNTR analysis, and revealed the population structure of *M. bovis* in Wales. Analyses uncovered three abundant, geographically distinct clusters. A further three clusters, each containing fewer isolates, were also geographically separated, with two of these showing particularly large SNP distances compared to most other Welsh isolates, suggesting independent introductions of *M. bovis* strains not endemic to Wales. WGS analysis provided enhanced resolution and clarified fine-scale population structure, offering important epidemiological insights into the population dynamics of *M. bovis* in Wales at both local and national levels. Finally, SNPs were identified in coding genes that have the potential for significant advantageous physiological consequences, which may impact host-pathogen interactions.

## Methods

### Sample preparation and sequencing

As part of the bTB statutory surveillance programme, all *M. bovis* culture-positive isolates collected during routine surveillance in 2021 were characterised by WGS. In multiple-reactor incidents only a sample of reactors are cultured, and generally no more than three animals with lesions are cultured per incident. Usually, only one culture-positive sample is collected from each incident for WGS. Samples were inoculated on modified 7H11 slopes^59^ for 6–12 weeks, and a single colony was harvested for molecular characterisation. These procedures were performed in Containment Level 3 (CL3) laboratories located at the Surveillance and Laboratory Services Department at APHA. Paired-end libraries were constructed directly from heat-inactivated material using the Nextera XT DNA Library Preparation Kit (Illumina, Cambridge, UK), and sequencing was performed at the Central Unit for Sequencing and PCR (CUSP) at APHA on an Illumina MiSeq or NextSeq 500/550 instrument, generating 150 bp paired-end reads.

### Bioinformatic analysis

Analysis was carried out using the bespoke Aberystwyth *M. bovis* pipeline (AMBoP) (v1.6.1), developed in-house for this analysis. The code is available on GitHub (github.com/Aber-TB/AMBoP). AMBoP is a pipeline that takes in raw, unprocessed reads, systematically uses tools to identify SNPs, and creates a variety of outputs, including trees and information on functional effects, whilst giving the user control over each parameter and step during the analysis. In brief, read quality assessments were undertaken before and after trimming using FASTQC^60^ and multiqc^61^. Sliding window quality trimming was used in Trimmomatic^62^. For trimming, a 10 bp window size and quality of 20 was used with a minimum read length of 36, and trimming adapters for various Illumina library preps were removed with 2 seed mismatches, 30 palindrome clip threshold and 10 simple clip thresholds (as per APHA parameters and as described in the GitHub repository btb-seq from APHA-CSU; https://github.com/APHA-CSU/btb-seq).

Paired trimmed reads were then aligned to a reference genome using BWA^63^ and unmapped, non-primary and supplementary alignments were removed using SAMtools^64^ as well as sorting, indexing and removing duplicates. For this study, the reference used was the *Mycobacterium bovis* AF2122/97 genome assembly (GenBank LT708304.1). Samples were retained if > 90% of the sites in the reference genome had a coverage depth of > = 10. Furthermore, read taxonomy was assigned using Kraken^65^. If < 70% of the raw reads from a sample were assigned ‘Mycobacterium’ in the taxonomy name but the sample passed the coverage and depth filters, then the taxonomy of reads that aligned to the reference genome were checked to determine the level of contamination (to evaluate whether non-Mycobacterial reads were aligned to the reference genome). This serves as a rescue step for samples that contain both Mycobacterial reads and contaminants, ensuring that only Mycobacterial reads are retained.

SNPs were called using BCFtools^64^ and resulting variant call files (vcf) were filtered; mapping quality (MQ) > = 30, depth of reads at site of alternative allele (DP) > = 10, number of supporting forward and reverse reads was > = 1 each, and proportion of reads needed to support an alternative allele was > = 0.95. For tree building, where a SNP was within 10 bp of other called variants, all were removed, as these loci can be associated with repetitive regions^66^ and/or are likely in linkage disequilibrium and can be indicative of non-neutrality in that genomic region. For returning functional information about SNPs these variants were retained. Sites within known hypervariable or repeat regions were masked and SNPs in these regions excluded, for example, those named as PE or PPE genes, labelled as direct repeats, invert repeats, repeat regions or mobile genetic elements in the annotation file for the genome available from GenBank (GFF3 available from NCBI Reference Sequence: NC_000962.3).

### Phylogenetic and population structure analyses

Maximum-likelihood phylogenies were constructed using the pseudo-genomes with IQ-tree^71^ using constant sites and with ultrafast bootstrapping (1000 replicates)^72^ and the best-fit model (TVM + F+I + I+R7) as identified by ModelFinder^73^. Optimal tree rooting was explored using a combination of the reference strain AF2122/97 and linages La1.1-1.7 of Zwyer et al.^11^. The resulting phylogenies was visualised and annotated in iTol^74^.

Assignment to the Mycobacterium lineages delineated by Zwyer et al.^11^., was conducted in TB-Profiler v6.6.6^75^. Spoligotypes were obtained using SpoTyping^76^ and the octal codes were queried in the *M. bovis* Spoligotype Database^77^. Isolates were further contextualised by assignment to the APHA WGS clade; this was achieved by constructing ML phylogenetic trees of the isolates from this study and those of Sandhu et al.^15^. Briefly, raw reads from these investigations were combined with Welsh isolates and run through the AMBoP pipeline, using all phylogenetic thresholds as described above, and ML trees were constructed using IQ-tree^71^ (parameters as stated above). Membership of each isolate to APHA WGS clade was defined based on phylogenetic clustering.

Analyses to further define population structure were conducted in R v2.4.2^78^. Briefly, concatenated SNP alignments were subjected to fast-hierarchical Bayesian Analysis of Population Structure (fast-BAPS)^79^ using a hierarchical Bayesian clustering algorithm^80^ and the optimised ‘symmetric prior’ to determine the optimal number of clusters at multiple levels of hierarchy. FastBAPs analysis was conducted using FastBAPs (version 1.0.8) and plotted with ggplot2^81^ and ggtree^82^, FastBAPS clustering was used to inform partitioning of clades into clusters on the phylogeny. Genetic distances between samples were explored using both the number of SNP differences and model-based genetic distances, calculated using ape^83^, and visualised using pheatmap (version 1.0.12) and ggtree in R v2.4.2^78^. Scripts for downstream data analysis and creation of figures can be found on GitHub (github.com/Aber-TB/AMBoP).

### Functional analysis of SNPs

Consensus FASTA files were created for each sample using the reference genome with alternative alleles in positions of predicted SNPs using BCFtools. SNPs present across all samples were identified using snp-sites^67^, creating a pseudo-genome (concatenated polymorphic sites) for each sample. SnpEff^68^ was used to predict the impact of SNPs and identify those located in functional genes. To get additional functional information, such as Clusters of Orthologous Genes (COGs), eggNOG-mapper online^69^ was used with default settings to annotate the protein-coding genes from the reference genome, obtained from NCBI.

Using the Eggnog annotations for the reference genome, SNPs that occurred in coding regions were grouped from samples within clusters and plotted using ggplot2, ggpubr^84^ and reshape2^85^ in R v2.4.2^78^. Counts of SNPs in genes were normalised using the gene length to account for the inherent likelihood that longer genes have more SNPs. Counts of SNPs in COG groups were normalised using the total number of genes in that COG group. Genes containing SNPs were also cross-referenced with lists of known virulence genes^24^ and gene essentiality^22^.

## Supplementary Information

Below is the link to the electronic supplementary material.

Supplementary Material 1

Supplementary Material 2

## Data Availability

Whole genome sequencing data were obtained from APHA under a data transfer agreement. Whole-genome sequencing data are deposited under project accession number PRJEB104470 (https://www.ebi.ac.uk/ena/browser/view/PRJEB104470), with the following bio-sample accession numbers: ERS28308286-ERS28308664 in the European Nucleotide Archive (ENA). The scripts for the analysis are made fully available online.
