# Supplementary material for: Uncovering the genomic landscape of Mycobacterium bovis in Wales
Source: Sci Rep. 2026 Sep 14;16:28546. doi: 10.1038/s41598-026-57014-2 (PMC13572552; doi:10.1038/s41598-026-57014-2)
Supplement: Supplementary file 1 [file 41598_2026_57014_MOESM2_ESM.docx]

# **Supplementary Material**

**Supplementary Figure 1:** **Maximum likelihood phylogenetic tree of *M. bovis* pseudo-genomes, inferred from 1,922 SNPs, from 2980 samples, including samples representative of the APHA WGS clades of Sandhu *et al* [15] as well as the 379 Welsh isolates from this study.** The coloured ring is colour-coded to represent the samples that belonged to: a) each WGS clade according APHA [15], and b) each Bayesian Analysis of Population Structure (BAPS) cluster. Clades are coloured based on assignment to each of the BAPS clusters delineated in this study. The tree was constructed in IQTree using the TVM+F+I+I+R7 model and rooted with Lineage La1.7.1 from Zwyer *et al.* [12].

**Supplementary Figure 2: Heatmap of the pairwise SNP distances, calculated as the number of SNPs that differ between *M. bovis* isolates.** Dark blue represents low numbers of SNPs that differ between samples and red represents a high number of SNPs. Samples are indicated based on their assignment to each of the genetic clusters identified by fastBAPS analysis.

**Supplementary Figure 3: Maximum likelihood phylogenetic tree of *M. bovis* pseudo-genomes, inferred from 1,971 SNPs, with the divergent clusters (BAPS clusters 1 & 2 in Figure 1) pruned from the tree.** The coloured rings are colour-coded to represent: i) the samples that belonged to each Bayesian Analysis of Population Structure (BAPS) cluster, ii) the samples that belonged to each BAPS level 2 (i.e., group clustering) cluster, and iii) the county in which the sample was isolated. The tree was constructed in IQTree using the TVM+F+I+I+R7 model and rooted with Lineage La1.7.1 from Zwyer *et al.* [12].

**Supplementary Figure 4: Phylogenetic placement of functionally relevant SNPs mapped on the Maximum likelihood phylogenetic tree of *M. bovis* pseudo-genomes, inferred from 1,971 SNPs from 379 isolates across Wales.** The coloured ring is colour-coded to represent the samples that belonged to each Bayesian Analysis of Population Structure (BAPS) cluster. Dots correspond to samples that contain key functional SNPs (i.e. those identified in multiple individuals), identified as being of high impact by SNPEff. The tree was constructed using IQTree, using model TVM+F+I+I+R7 and rooted with Lineage La1.7.1 from Zwyer *et al.* [12].

**Supplementary Table 1: Pairwise SNP differences between each of the clusters identified by fastBAPS analysis.** Displayed as the average number of SNP that differ between the members of each cluster below the diagonal and the standard deviation above the diagonal.

| **Cluster** | 1 | 2 | 3 | 4 | 5 | 6 |
| --- | --- | --- | --- | --- | --- | --- |
| 1 |  | 2.45 | 4.04 | 9.46 | 4.40 | 4.13 |
| 2 | 475.90 |  | 8.07 | 12.13 | 8.26 | 8.13 |
| 3 | 416.82 | 516.09 |  | 16.29 | 4.55 | 4.22 |
| 4 | 408.16 | 507.13 | 140.93 |  | 18.16 | 21.32 |
| 5 | 406.34 | 505.64 | 156.22 | 140.26 |  | 4.52 |
| 6 | 410.88 | 510.15 | 160.87 | 142.83 | 122.51 |  |

**Supplementary Table 2: The minimum-maximum number of SNPs that differ between each of the clusters identified by BAPS analysis.**

| **Cluster** | 1 | 2 | 3 | 4 | 5 |
| --- | --- | --- | --- | --- | --- |
| 1 |  |  |  |  |  |
| 2 | 471-480 |  |  |  |  |
| 3 | 407-430 | 500-532 |  |  |  |
| 4 | 394-427 | 487-529 | 109-170 |  |  |
| 5 | 394-419 | 487-521 | 138-175 | 98-172 |  |
| 6 | 398-423 | 491-523 | 142-179 | 95-176 | 106-140 |

**Supplementary Table 3: Number of SNP differences within the six clusters identified by BAPS analysis.** Given as mean number of SNPs that differ between members of that cluster and its standard deviation (stdev) and the minimum and maximum numbers of SNPs that differ between any members of that cluster. N= number of samples assigned to that cluster.

| cluster | N | Mean | StDev | Min | Max |
| --- | --- | --- | --- | --- | --- |
| 1 | 7 | 21.10 | 3.09 | 16 | 26 |
| 2 | 3 | 23.330 | 15.04 | 6 | 33 |
| 3 | 65 | 17.66 | 6.89 | 0 | 42 |
| 4 | 8 | 129.32 | 44.22 | 5 | 166 |
| 5 | 118 | 23.39 | 7.86 | 0 | 44 |
| 6 | 179 | 17.48 | 10.43 | 0 | 64 |

**Supplementary Table 4: Count of SNPs occurring in COG functional categories for each cluster.** The counts of genes within each category in the reference is given for context and to allow for normalisation of the counts.

|  | Cluster | | | | | |  |  |  |
| --- | --- | --- | --- | --- | --- | --- | --- | --- | --- |
| COG group | **1** | **2** | **3** | **4** | **5** | **6** | **Ref** | **Total** | **Total adjusted** |
| A | 0 | 0 | 0 | 0 | 0 | 0 | 2 | 0 | 0 |
| B | 0 | 0 | 3 | 0 | 0 | 0 | 1 | 3 | 3 |
| C | 183 | 99 | 543 | 59 | 770 | 1084 | 242 | 2738 | 11.31405 |
| D | 56 | 18 | 269 | 17 | 255 | 371 | 56 | 986 | 17.60714 |
| E | 165 | 96 | 538 | 70 | 1095 | 1833 | 233 | 3797 | 16.29614 |
| F | 85 | 22 | 242 | 12 | 265 | 549 | 109 | 1175 | 10.77982 |
| G | 123 | 60 | 387 | 38 | 395 | 743 | 138 | 1746 | 12.65217 |
| H | 98 | 60 | 211 | 33 | 250 | 583 | 163 | 1235 | 7.576687 |
| I | 231 | 100 | 853 | 66 | 820 | 1374 | 329 | 3444 | 10.46809 |
| J | 95 | 43 | 74 | 13 | 245 | 752 | 178 | 1222 | 6.865169 |
| K | 187 | 102 | 597 | 62 | 1185 | 1993 | 289 | 4126 | 14.27682 |
| L | 169 | 98 | 675 | 71 | 730 | 911 | 225 | 2654 | 11.79556 |
| M | 113 | 57 | 523 | 44 | 706 | 716 | 144 | 2159 | 14.99306 |
| N | 21 | 9 | 0 | 0 | 0 | 1 | 81 | 31 | 0.382716 |
| O | 56 | 31 | 136 | 15 | 43 | 198 | 106 | 479 | 4.518868 |
| P | 171 | 107 | 464 | 53 | 641 | 983 | 173 | 2419 | 13.98266 |
| Q | 152 | 115 | 683 | 42 | 814 | 660 | 241 | 2466 | 10.23237 |
| T | 78 | 50 | 357 | 31 | 436 | 582 | 117 | 1534 | 13.11111 |
| U | 28 | 6 | 5 | 2 | 6 | 12 | 36 | 59 | 1.638889 |
| V | 49 | 36 | 197 | 14 | 8 | 76 | 69 | 380 | 5.507246 |
| S | 447 | 253 | 1947 | 216 | 2102 | 4496 | 885 | 9461 | 10.6904 |
| - | 163 | 62 | 497 | 43 | 776 | 1466 | 294 | 3007 | 10.22789 |
| no COG | 44 | 12 | 9 | 10 | 467 | 163 | 223 | 705 | 3.161435 |
